# Characterization of Spleen Transcriptome and Immunity Against Avian Colibacillosis After Immunization With Recombinant Attenuated *Salmonella* Vaccine Strains

**DOI:** 10.3389/fvets.2018.00198

**Published:** 2018-08-21

**Authors:** Zachary R. Stromberg, Angelica Van Goor, Graham A. J. Redweik, Melha Mellata

**Affiliations:** Department of Food Science and Human Nutrition, Iowa State University, Ames, IA, United States

**Keywords:** avian pathogenic *Escherichia coli*, colibacillosis, ExPEC, poultry, *Salmonella* vaccine, transcriptome

## Abstract

Avian pathogenic *Escherichia coli* (APEC) causes extraintestinal infections in poultry. Vaccines targeting APEC in chickens have been partially successful, but many lack heterologous protection. Recombinant attenuated *Salmonella* vaccine (RASV) strains can induce broad immunity against *Salmonella* and be modified to deliver *E. coli* antigens. Along with vaccine characteristics, understanding the host response is crucial for developing improved vaccines. The objectives of this study were to evaluate host responses to vaccination with an RASV producing *E. coli* common pilus (ECP) and assess protection against APEC infection in chickens. Four-day-old White Leghorn chickens were unvaccinated or orally vaccinated and boosted 2 weeks later with RASV χ8025(pYA3337), RASV χ8025(pYA4428) carrying *ecp* operon genes, or a combination of χ8025(pYA3337) and χ8025(pYA4428) (Combo). To assess host responses, serum IgY and intestinal IgA antibody titers were measured, and spleen samples (*n* = 4/group) were collected from unvaccinated and Combo vaccinated 4-week-old chickens for RNA-seq. Vaccine protection potential against *Salmonella* and APEC was evaluated *in vitro* using bacterial inhibition assays. Five-week-old chickens were challenged via air sac with either an APEC O2 or O78 strain. *E. coli* was enumerated from internal organs, and gross colibacillosis lesions were scored at necropsy. RASV immunized chickens elicited anti-*E. coli* antibodies. The spleen transcriptome revealed that 93% (89/96) of differentially expressed genes (DEG) were more highly expressed in Combo vaccinated compared to unvaccinated chickens, with signal as the most significantly impacted category. RNA-seq analysis also revealed altered cellular and metabolic processes, response to stimulus after vaccination, and immune system processes. Six DEG including genes linked to transcription regulation, actin cytoskeleton, and signaling were highly positively correlated with antibody levels. Samples from RASV immunized chickens showed protection potential against *Salmonella* strains using *in vitro* assays, but a variable response was found for APEC strains. After APEC challenges, significant differences were not detected for bacterial loads or gross lesions scores, but χ8025(pYA3337) immunized and χ8025(pYA4428) immunized chickens had significantly fewer number of APEC-O2-positive samples than unvaccinated chickens. This study shows that RASVs can prime the immune system for APEC infection, and is a first step toward developing improved therapeutics for APEC infections in chickens.

## Introduction

Avian pathogenic *Escherichia coli* (APEC) is a subset of extraintestinal pathogenic *E. coli* (ExPEC) that commonly causes respiratory and systemic infections in poultry. APEC poses a threat to poultry health (1, 2); an estimated 60–70% of total mortality is associated with APEC infection in flocks of young chickens (3, 4). Humans can also acquire APEC through direct contact with animals or contaminated poultry meat and eggs, and these organisms have the potential to cause severe ExPEC-associated human infections (2, 5–8). Reduction of APEC in food-producing animals may have the potential to reduce pathogen spread between animals and the number of human ExPEC infections (6, 9).

Commonly, chickens acquire APEC through a respiratory route of infection, leading to septicemia, fibrinous lesions of internal organs, and death. APEC strains also cause local infections in poultry, including cellulitis, salpingitis, and synovitis (1, 10). APEC-infected chickens result in economic losses to the poultry industry because of lost production time, containment, carcass condemnations, and mortality. Due to antimicrobial resistance and restriction of antibiotic usage, management of APEC infections is challenging (1, 11). Moreover, changes in poultry production practices including increases in free-range production may lead to greater incidence of avian colibacillosis (12). A prevention strategy to control disease would have the potential for significant benefits for poultry and producers.

Current vaccine strategies to prevent APEC infections in chickens have been only partially successful at preventing disease and mortality. In one study, vaccination with a live attenuated *E. coli* O78 strain was protective against O78 APEC challenge and provided some protection against gross lesions when challenged with a non-lethal untypeable APEC strain (13). Other studies have focused on polysaccharide vaccines that have provided protection against O1 and O2 APEC serogroups (14, 15). Although serogroups O1, O2, and O78 are often identified in poultry infections, other serogroups have been associated with disease (1, 10). Thus, vaccines containing only serogroup specific factors may not prevent infection of heterologous APEC strains. Targeting shared factors of APEC may prevent infection of multiple APEC serogroups. Thus, *E. coli* common pilus (ECP) was selected as a vaccine antigen, because from our previous study, it was found in 76% (127/167) of APEC strains of various serotypes and plays a role in APEC virulence (16). The *ecp* operon encodes for surface proteins including major pilin protein EcpA and tip pilus adhesin protein EcpD (17). To deliver ECP, a recombinant attenuated *Salmonella* vaccine strain (RASV) was used. RASV strains were successful in reducing *Salmonella* in chickens in previous studies and can be modified to provide protection against other pathogens (18–22).

Both bacterial and host factors contribute to the severity of APEC infection (10). Therefore, understanding host responses to vaccination is needed to generate improved therapeutics to combat infection. The spleen was selected to study host response because it is a major immune organ in chickens that responds to infections and foreign antigens (10, 23). Overall, the objectives of this study were to (i) evaluate the serum and mucosal antibody responses elicited in chickens vaccinated orally with RASVs; (ii) characterize the transcriptome response of spleens from chickens immunized with a combination of RASVs; and (iii) assess the protective capability of RASVs using *in vitro* assays and an avian colibacillosis challenge.

## Materials and methods

### Chickens

One-day-old female and male, specific pathogen-free, White Leghorn chickens were obtained from Valo BioMedia (Adel, IA), and acclimated for 3 days before any experiments began. Chickens received enrichment devices and were housed in floor pens containing deep wood shavings. Throughout the study, chickens had *ad libitum* access to feed and water. Animals were individually identified throughout the study using leg bands. Humane endpoint criteria were set for all chickens such that any moribund animal or animals exhibiting immobility (unable to feed or drink) were euthanized by CO_2_ inhalation according to recommendations of the American Veterinary Medical Association Guidelines for Euthanasia, 2013 edition.

### Bacterial strains, plasmids, and growth conditions

Strains and plasmids used are listed in Table 1. In the Δ*asd* attenuated *Salmonella enterica* serotype Typhimurium strain χ8025 (25), a derivative of the virulent *S*. Typhimurium strain χ3761 (24), the Asd^+^ (aspartate β-semialdehyde dehydrogenase) plasmids pYA3337 (empty plasmid) or pYA4428 that carries the *ecp* operon (29) were introduced as previously described (29). Safety in chickens has been shown with RASV strains containing the same Δ*cya* Δ*crp* mutations used in the current study (24). These mutations also eliminate the catabolite repressor protein, which allows ECP to be constitutively expressed (25). APEC strains APEC-O2 (O2:K2) (27) and χ7122 (O78:K80:H9) (26) were used for animal infection experiments. Although both APEC strains carry genes for *ecp*, we found previously that APEC-O2 was positive for ECP synthesis *in vitro*, but χ7122 tested negative (16). *Salmonella* strains χ3761, AR-404, AR-408, AR-409, and CVM29188 and APEC strains χ7122, χ7503 (O1), and APEC-O2 were used for *in vitro* assays. Stock cultures were stored at −80°C in trypticase soy broth containing 15% glycerol. RASV and challenge *E. coli* strains were grown statically overnight in 3 ml of lysogeny broth (LB) at 37°C. The next day, the culture was inoculated 1:50 into fresh LB at 37°C using a flask-to-medium ratio of 5:1. RASV strains were grown with aeration to OD_600_ of ~0.85. APEC challenge strains were grown statically overnight. Strains were harvested by centrifugation at 22°C and resuspended in PBS.

**Table 1:** Bacterial strains and plasmids.

**Strain or plasmid**	**Relevant genotype, phenotype, and/or characteristics**	**References^a^**
***Salmonella enterica***		
χ3761	*S*. Typhimurium UK-1	(24)
χ8025	*S*. Typhimurium, Δ*cya-27 Δcrp-27 ΔasdA16*	(25)
AR-404	*S*. Heidelberg	CDC
AR-408	*S*. Typhimurium	CDC
AR-409	*S*. Typhimurium	CDC
CVM29188	*S*. Kentucky	
***Escherichia coli***		
χ7122	Avian pathogenic *E. coli* O78:K80:H9	(26)
χ7503	Avian pathogenic *E. coli* O1	(16)
APEC-O2	Avian pathogenic *E. coli* O2:K2	(27)
**PLASMID**		
pYA3337	*asd*-based cloning vector (pSC101 *ori*) with P*_*trc*_* promoter	(28)
pYA4428	The *ecpABCD* was cloned under P*_*trc*_* of pYA3337	(29)

a *CDC, United States Centers for Disease Control and Prevention*.

### Construction of RASV χ8025 containing *ecp* genes and evaluation of ECP synthesis

The previously constructed *asd*-positive ECP expression plasmid pYA4428 was evaluated for ECP synthesis in χ8025 by transmission electron microscopy using rabbit anti-EcpA and -EcpD antibodies as described earlier (29).

### Oral immunization and antibody responses

One-day-old chickens were randomly divided into four groups (*n* = 22–26/group) and housed in isolated rooms. At 4-days-old, chickens were orally administered 20 μl of PBS (unvaccinated) or a total of 10^8^ colony-forming units (CFU) of χ8025(pYA3337), or χ8025(pYA4428), or a combination of equal parts χ8025(pYA3337) and χ8025(pYA4428) (hereafter referred to as Combo) via pipette. Water and food were removed 4 h before and resupplied 30 min after vaccination. Previously, both RASV strain χ9558(pYA3337) lacking *ecp* and χ9558(pYA4428) with *ecp* protected against human ExPEC-associated disease in mouse models, but the amount of protection found in different organs depended on the strain (29). Thus, the Combo vaccine was used to test whether the mixture of RASV strains provided superior protection against APEC infection in the present study. Chickens were boosted 2 weeks later (18-days-old) using the same dose and procedures. Serum and intestinal wash samples were collected from 32-day-old chickens. Blood was obtained from the wing vein of all chickens and centrifuged at 1,000 × g for 10 min to isolate serum. For intestinal wash samples, chickens were euthanized (*n* = 4/group) and intestinal wash samples were collected as described previously (30). Serum and intestinal wash samples were stored at −80°C until further use. Bursa tissue and cecal contents were collected from euthanized chickens to assess RASV colonization level. For enumeration of RASV strains, samples were weighed, serially diluted in PBS, plated on MacConkey agar, and plates were incubated at 37°C for 24 h. After incubation, lactose-negative colonies with typical morphology of RASV strains were quantified and converted to CFU/g.

Serum IgY and intestinal wash IgA antibodies against EcpA or iron-uptake receptors (IroN and IutA) were detected by ELISA using *E. coli* antigens as prepared previously (29, 31). Briefly, genes encoding EcpA, IroN, and IutA were amplified and cloned into pET-101/D-TOPO, expressed in *E. coli* BL21, and His-tagged proteins were purified using standard methods including nickel columns and endotoxin removal spin columns (Pierce Biotechnology, Rockford, IL). Plates were coated with 2.0 μg/ml of proteins and incubated overnight at 4°C. The remaining steps were performed at room temperature. Plates were washed with PBS containing 0.05% Tween-20 and blocked for 1 h with SEA BLOCK (Thermo Scientific). Serum at 1:50 or intestinal wash samples at 1:10 were added and diluted 2-fold down the plate. After 1 h incubation, goat anti-chicken IgY, alkaline phosphatase conjugate for serum (1:5,000; ThermoFisher Scientific) or goat anti-chicken IgA, alkaline phosphatase conjugate for intestinal wash samples (1:5,000; Abcam, Cambridge, MA) was added and plates were incubated for 1 h. Plates were developed using p-nitrophenyl phosphate. Reactions were stopped with 2 N NaOH and plates were read at 405 nm. Endpoint titer was defined as the reciprocal of the highest dilution that gave an OD_405_ twice that of the unvaccinated group.

### Spleen collection, RNA extraction, library generation, and sequencing

Immediately after euthanasia, spleen samples were collected from unvaccinated and Combo vaccinated 32-day-old chickens (*n* = 4/group), placed in RNAlater™ (Ambion) for 24 h at room temperature, and then stored at −80°C until further use. RNA was isolated using the RNeasy® Mini Kit (Qiagen) following manufacturer's instructions. RNA quality was determined using the Agilent 2100 Bioanalyzer and samples with an RNA integrity number (RIN) >9 were used for RNA-seq. Generation of cDNA libraries from RNAs were constructed using the TruSeq RNA Library preparation kit v2 (Illumina) which selectively amplifies PolyA mRNA. All samples were individually barcoded, multiplexed, and sequenced on a single lane on the HiSeq 3000 (Illumina) generating 150 bp single-end reads.

### RNA-seq analysis, detection of differential gene expression, and functional annotation

Quality control of RNA-seq data was completed using the programs FASTX Clipper and Quality Trimmer with the following options: sequences shorter than 25 nucleotides were discarded, a minimum phredd score of 25, and adapter sequences were removed. Tophat (version 2.1.1) (32) was used for alignment to the *Gallus gallus* genome (version 5.0 with 5.0.86 GTF Ensembl) using default parameters. Mapped reads were counted to genes using HTSeq (version 3.0). Differentially expressed genes (DEG) were detected using EdgeR (version 2.0), with the Benjamini-Hochberg method implemented for multiple testing correction, and maximum false discovery rate (FDR) ≤ 0.05. The cutoff value for expressed genes was 1 FPKM. A pairwise comparison between unvaccinated and Combo immunized chickens was used to detect DEG. To calculate the percentage of expressed genes, the number of genes with counts ≥1, determined by HTSeq, was divided by the total number (14,468) of annotated genes in the Galgal5 genome. Visualization of DEG was completed using clustering analysis and a volcano plot using SARTools software with default parameters (33).

For functional annotation, DAVID Bioinformatics Resources^1^ (version 6.8) was used with the option of functional annotation clustering using input of 96 DEG (cutoff adjusted *P*-value ≤ 0.05) with default parameters and Benjamini-Hochberg multiple testing *P*-value adjustment. Panther Database^2^ (version 12.0) was used for the functional classification viewed in pie chart analysis using input of 96 DEG (cutoff adjusted *P*-value ≤ 0.05) with default parameters with the ontology options molecular function and biological process implemented. Protein-protein interaction networks were created in the program STRING (34) with default parameters using the DEG list as input data. Only genes with one or more connections to other genes were included. To link host immunity to gene expression, the Combo vaccine group serum IgY antibody responses against EcpA, IroN, and IutA were correlated to the normalized DEG counts using JMP (JMP®, Version 12.0.1. SAS Institute Inc., Cary, NC) for the four animals used in RNA-seq. The correlations were used to generate a heatmap in JMP for visualization.

RNA-seq data in this publication are deposited in the Gene Expression Omnibus at NCBI^3^ (35) with the GEO accession number GSE101198.

### The effect of vaccination on commensal *E. coli* in chickens

Freshly voided fecal samples (*n* = 3/group) were collected weekly. For enumeration of *E. coli*, samples were weighed, serially diluted in PBS, plated on MacConkey agar, and plates were incubated at 37°C for 18 h. After incubation, *E. coli* (lactose-positive colonies) was quantified and converted to CFU/g of feces.

### Serum and intestinal wash bacterial inhibition assays

Inhibition of bacterial strains in pooled serum (*n* = 22–26/group) or intestinal wash samples (*n* = 4/group) obtained from 32-day-old chickens was performed as described previously with minor modifications (29). Bacterial strains were grown overnight on LB agar at 37°C. The next day, colonies were suspended in M9 minimal media until OD_600_ reached 1.0. The bacterial suspension was diluted in M9 minimal media and 10^2^ CFU was mixed 1:9 with pooled serum or intestinal wash samples and incubated at 40°C for 6 h. After incubation, the mixture was serially diluted in PBS, plated on MacConkey agar, and then quantified and converted to CFU/ml. Three independent inhibition assays were performed with each assay done in triplicate.

### Air sac challenge

The left caudal thoracic air sac of chickens (37-days-old) was inoculated with 10^7^ CFU of either APEC-O2 or χ7122. All groups had at least 8 chickens and groups were housed in separate rooms. At 24 h post-challenge, blood was collected, and at 48 h post-challenge chickens were euthanized by CO_2_ inhalation. Heart, liver, lung, and spleen tissues, and an air sac swab were collected for detection and enumeration of *E. coli* using MacConkey agar. Gross lesion scoring for colibacillosis inflammation on the air sac and combined scoring for heart and liver were performed as described previously (36).

### Statistical analysis

An ANOVA followed by Tukey's test for multiple means comparison was used to compare between groups for RASV colonization, ELISA, *E. coli* shedding, *in vitro* bacterial inhibition assays, and bacterial loads in APEC infection experiments. Fisher's exact test (two-tailed) was used to compare unvaccinated and vaccinated chickens for the proportion of tissues positive for *E. coli* in APEC infection experiments. Analyses were performed using GraphPad Prism 6.0. *P*-values < 0.05 were considered statistically significant.

## Results

### ECP synthesis in RASV χ8025(pYA4428)

As screened by transmission electron microscopy, ECP production was detected on the surface of χ8025(pYA4428) and positive control strain CFT073, but not on χ8025(pYA3337) as expected (Figure S1).

### RASV colonization and antibody induction

To determine if RASV strains colonized chickens, bursa tissue and cecal contents were collected from 32-day-old chickens (Figure S2). As a control, the unvaccinated birds were tested and confirmed to be RASV-negative. No significant differences were detected for RASV colonization of the bursa. RASV strains were recovered from bursa tissue of 1 of 4 χ8025(pYA3337) immunized chickens, 2 of 4 Combo immunized chickens, and were not detected in χ8025(pYA4428) immunized chickens. For the cecal contents, Combo immunized birds had significantly higher levels of RASV strains compared to other groups and could be recovered from all 4 birds. However, whether both or only one RASV strain was colonizing the Combo birds was not differentiated.

**Figure 1:**
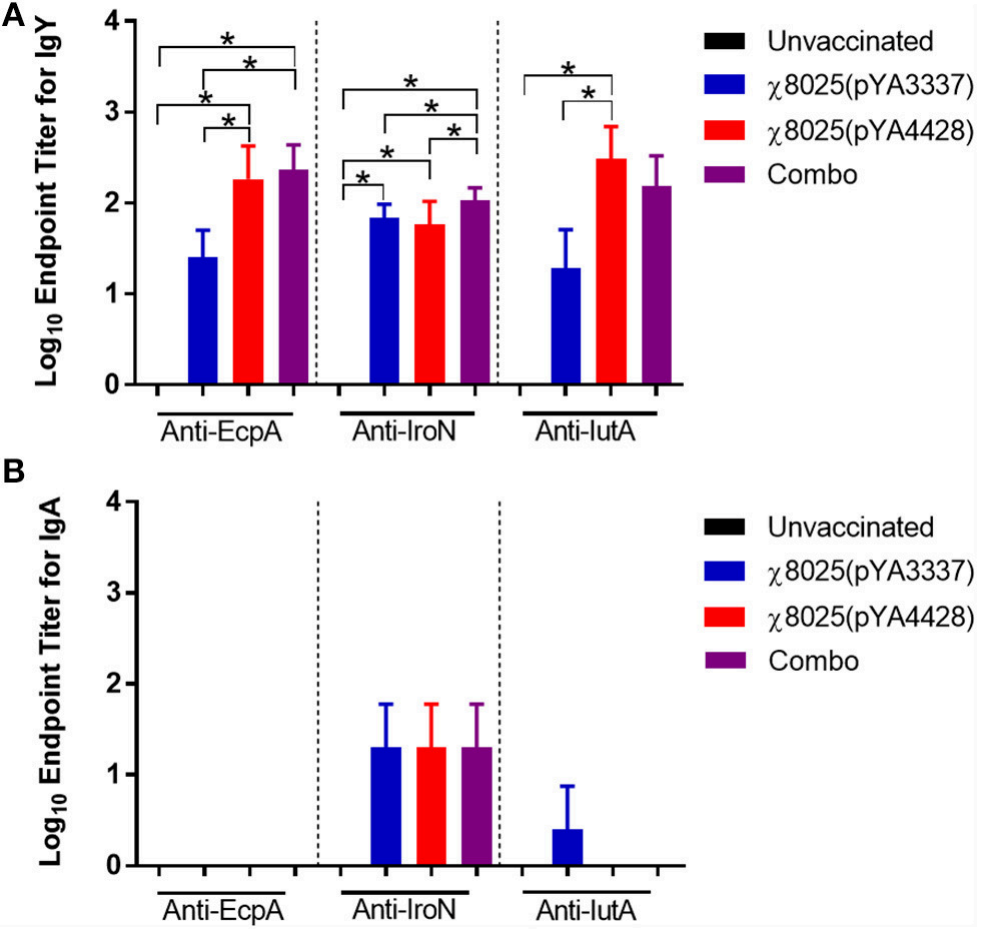
Antibody responses of unvaccinated and vaccinated chickens against specific *Escherichia coli* antigens. Data represent **(A)** serum IgY and **(B)** intestinal IgA antibody levels induced in chickens immunized with either PBS, χ8025(pYA3337), χ8025(pYA4428), or Combo referring to the combination of χ8025(pYA3337) and χ8025(pYA4428). χ8025(pYA3337) and χ8025(pYA4428). Individual sera from 22-26 chickens per group and intestinal wash sample from 3-4 chickens per group were analyzed by ELISA against *E. coli* antigens EcpA, IroN, and IutA. An asterisk (*) represents a statistically significant difference (*P* < 0.05) as determined by an ANOVA followed by Tukey's test for multiple means comparison. Bars represent the mean and error bars represent standard deviations.

Results for serum IgY and intestinal wash IgA antibodies against *E. coli* antigens are shown in Figure 1. Anti-EcpA IgY antibodies were detected in 32-day-old RASV immunized chickens and were significant for χ8025(pYA4428) and the Combo vaccine compared to unvaccinated and χ8025(pYA3337) immunized chickens. Significantly greater levels of anti-IroN IgY antibodies than observed for unvaccinated chickens were elicited in all RASV immunized chickens. Additionally, anti-IroN IgY antibody titers of Combo vaccinated chickens exceeded those for individual χ8025(pYA3337) or χ8025(pYA4428) immunized chickens. Anti-IutA IgY antibody titer of χ8025(pYA4428) immunized chickens was greater than that of unvaccinated and χ8025(pYA3337) immunized chickens, but not significantly different from the Combo vaccine. For intestinal wash samples, anti-IroN IgA antibodies were detected at a similar level for RASV immunizations, but each one was not significantly different from unvaccinated antibody titers. No anti-EcpA IgA antibodies were detected and only one chicken immunized with χ8025(pYA3337) elicited a detectable response for anti-IutA IgA antibodies.

### RNA-seq quality control, alignment, and mapping to the chicken genome

**Figure 2:**
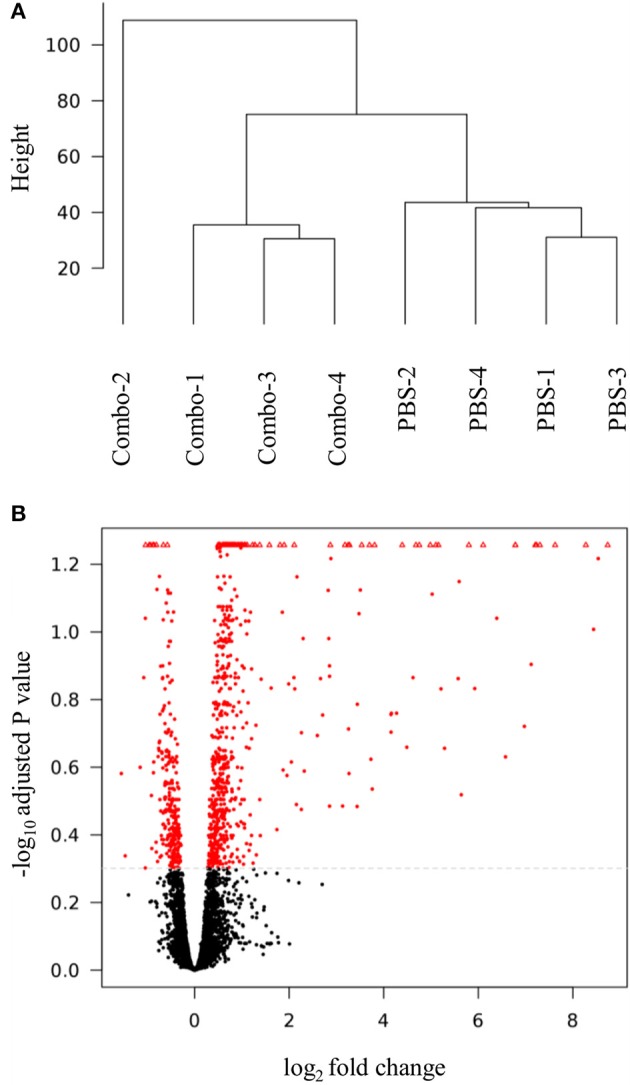
Differential gene expression analysis of spleen samples. The **(A)** dendrogram cluster and **(B)** volcano plot were generated in SARTools. Combo refers to the vaccine of χ8025(pYA3337) and χ8025(pYA4428).

Table S1 reports RNA-seq data pre- and post-filtering generated from the spleens of unvaccinated and Combo vaccinated chickens. The mean number of sequences generated pre- and post-filtering were 48,574,449 and 40,594,598 sequences per sample, respectively. The mean percentage of expressed genes was 89.6%. Visualization of DEG in a dendrogram cluster and a volcano plot are depicted in Figure 2. The dendrogram results (Figure 2A) show the four samples from unvaccinated chickens clustered together, whereas three of four Combo vaccinated samples clustered together. In the volcano plot, most genes did not meet the differential gene expression cutoff values (black) (Figure 2B). Almost all genes colored red and considered significant are increased in expression within the Combo vaccinated group i.e., positive log_2_ fold change and those with a negative value did not deviate far from zero i.e., >−2 log_2_ fold change.

### Vaccination with combo results in DEG and enriched functional categories in the spleen

In spleen samples, DEG were determined as those with an FDR ≤ 0.05 cutoff for significance and are reported in Table S2 in order of fold change. A total of 96 genes were identified as differentially expressed with 90 annotated genes and 6 genes classified as uncharacterized. Most DEG (89/96) were more highly expressed in the Combo vaccinated compared to the unvaccinated group. In the Combo vaccinated group fold changes ranged between 1.4 and 426.3 (higher expression) and between 0.5 and 0.7 (lower expression), compared to the unvaccinated group.

**Figure 3:**
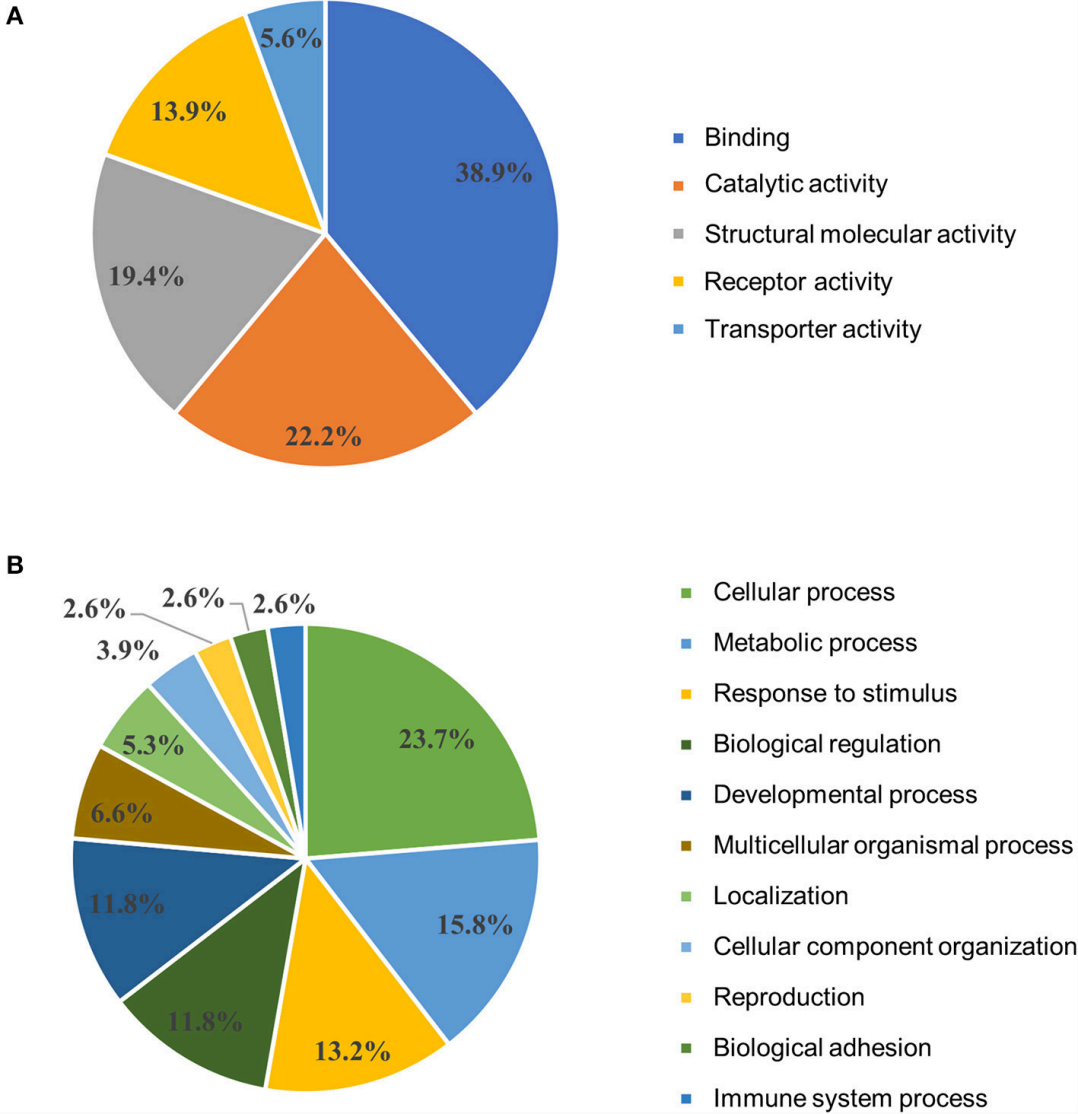
Gene ontology clustering analysis. Differentially expressed genes from the comparison of unvaccinated to Combo vaccinated chickens were analyzed in Panther based on their **(A)** molecular function and **(B)** biological process. Combo refers to the vaccine of χ8025(pYA3337) and χ8025(pYA4428).

DAVID functional annotation clustering analysis resulted in 67 out of 96 input DEG annotated within the software. Six clusters significantly (adjusted *P* < 0.05) impacted are presented in Table 2. Signal was the most significantly (lowest *P*-value) impacted with 26 genes in this category. Additional clusters included disulfide bond, glycoprotein, extracellular matrix, immunoglobulin domain, and muscle protein ranging from 5 to 15 genes in each category. Results from the gene ontology (GO) enrichment analysis resulted in 54 out of 96 input DEG annotated within the software and are depicted in Figure 3. The molecular function chart (Figure 3A) revealed five categories of enrichment with our input. Binding and catalytic activity together accounted for 61.1%. The remaining categories included structural molecular activity, receptor activity, and transporter activity. The biological process chart (Figure 3B) revealed 11 categories of enrichment within our gene list. Cellular process, metabolic process, and response to stimulus together accounted for 52.7%. Other categories of interest included biological adhesion and immune system process accounting for 2.6% each.

**Table 2:** Functional annotation clustering of differentially expressed genes.

**Category**	**No. of genes**	**Adjusted *P*-value**	**Gene names**
Signal	26	4.9E-4	*RSPO1, THY1, CHRD, C1HXORF36, C4orf48, CNTFR, CCDC80, COL6A1, COL6A3, LOC419851, EDN2, FGFBP1, FBLN5, FSTL1, FRZB, ISLR, LCAT, LL, MXRA8, NTN3, OC3, PODXL, PIGR, SPARC, SERPINF1, SFTPA*
Disulfide bond	15	8.4E-3	*THY1, CNTFR, COL6A3, LOC419851, FBLN5, FRZB, ISLR, LCAT, LL, MXRA8, NTN3, OC3, SPARC, SFTPA, TIE1*
Glycoprotein	10	2.6E-2	*THY1, CNTFR, COL6A1, COL6A3, FRZB, LCAT, MXRA8, NTN3, PODXL, SPARC*
Extracellular matrix	5	2.1E-2	*CCDC80, COL6A1, COL6A3, NTN3, SPARC*
Immunoglobulin domain	5	3.4E-2	*THY1, CNTFR, ISLR, MXRA8, TIE1*
Muscle protein	5	2.0E-3	*ACTA1, ACTA2, MYH11, MYL9, TAGLN*

**Figure 4:**
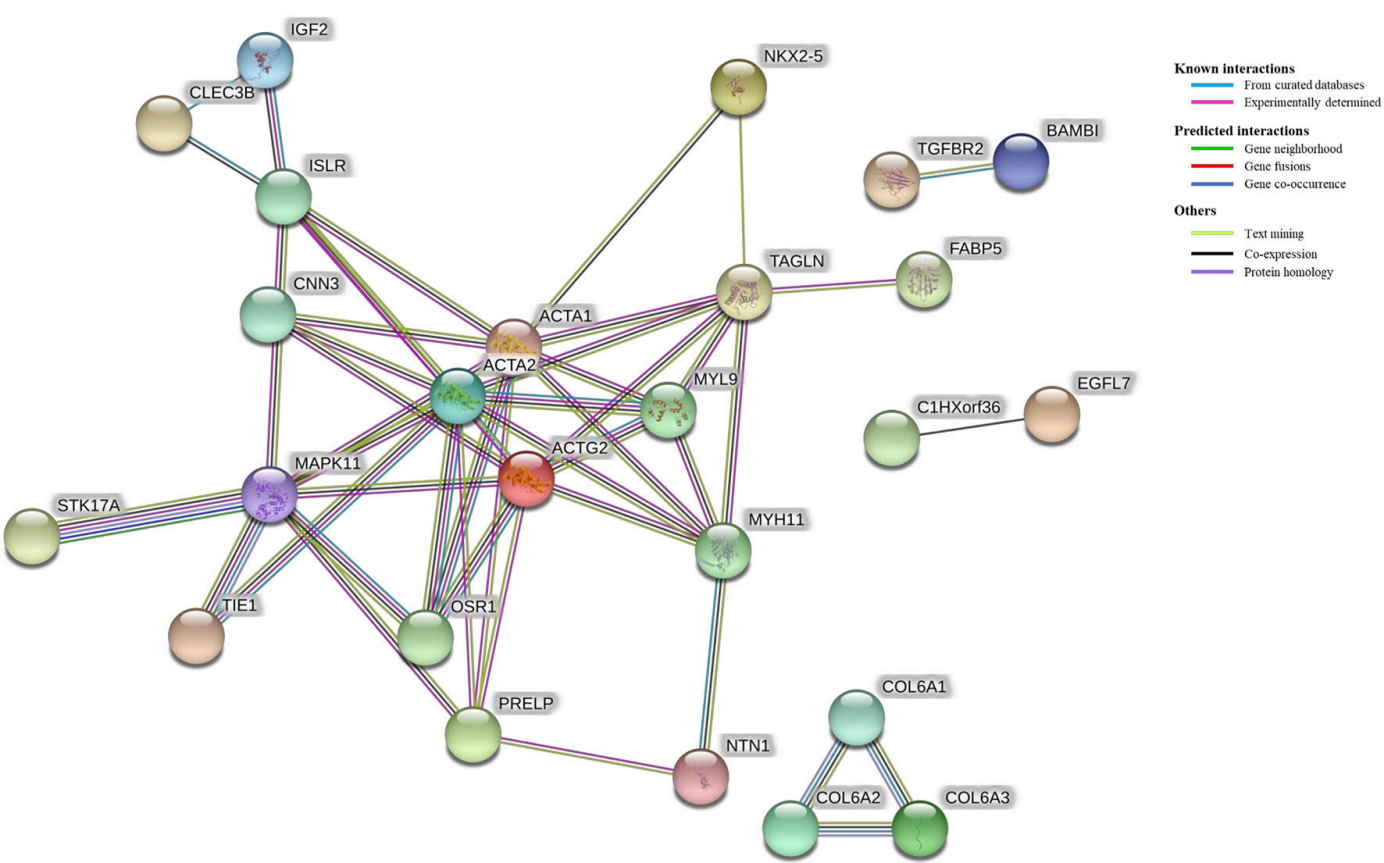
Protein-protein interaction network among differentially expressed genes (DEG). All of the DEG (*N* = 96) from the comparison of unvaccinated vs. Combo were used as input in a protein-protein interaction network analysis. Edges connecting proteins indicate evidence of association and are colored based on the type of interaction including known, predicted, and other. Only DEG that had one or more interactions are displayed (*n* = 25).

The results of the protein-protein interaction network analysis are found in Figure 4. The comparison of unvaccinated vs. Combo revealed connectivity between 25 of 96 DEGs. The strongest clustering was between genes that function in cell motility including ACTA1, ACTA2, ACTG2, CNN3, MYH11, MYL9, and TAGLN. The MAPK11 protein was a hub having 8 connections with other proteins.

**Figure 5:**
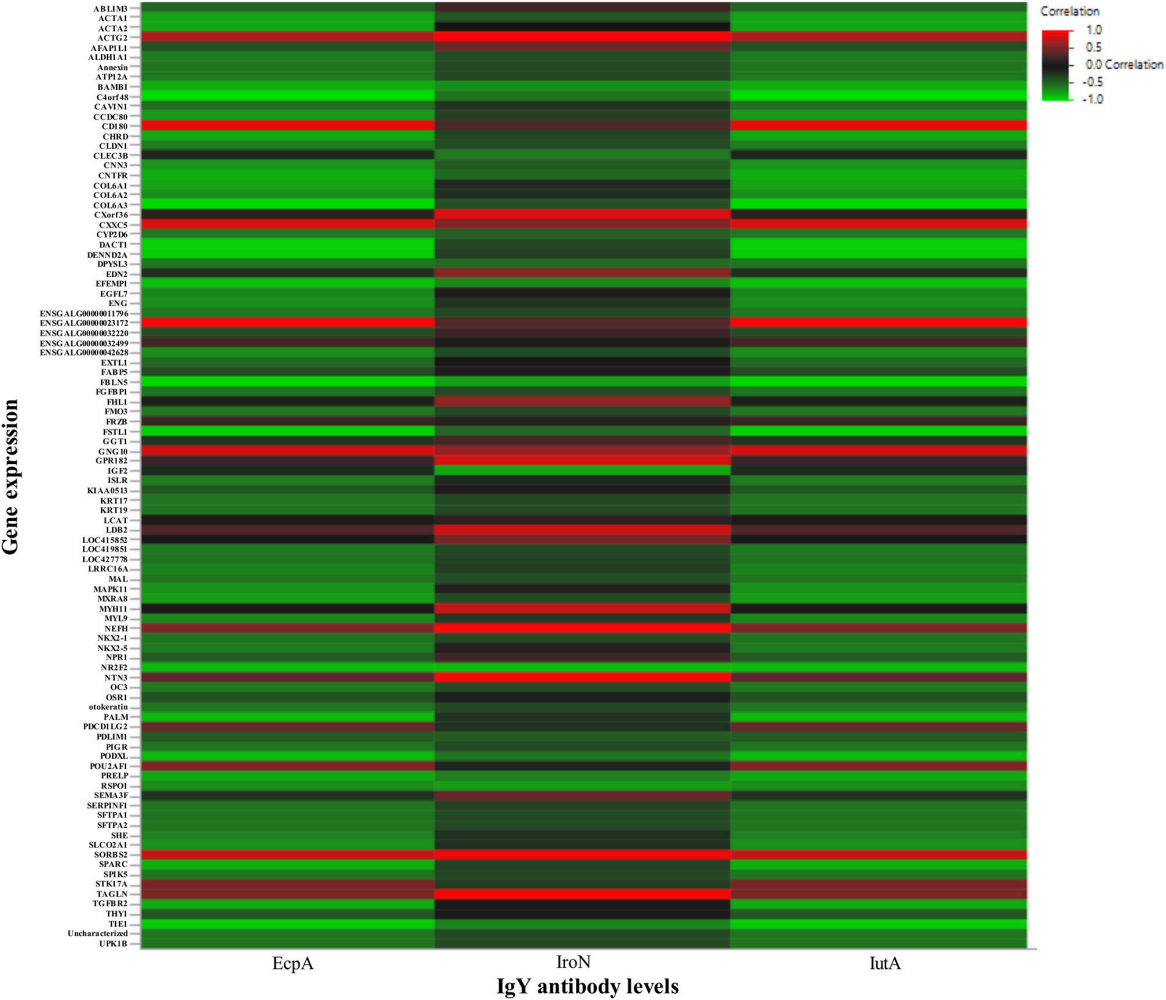
Heatmap of the correlation between antibody response and gene expression. The heatmap was generated using correlations between serum IgY antibody levels in Combo measuring EcpA, IroN, and IutA (x-axis) with normalized gene expression levels of DEG (*N* = 96) in Combo (y-axis). The color and intensity represent the *R*^2^ correlation coefficient ranging from 1 to −1 from red to green, respectively.

The results of the heatmap based on correlations between serum IgY anti-EcpA, IroN, and IutA antibody response to vaccination with the normalized DEG counts in the Combo group can be found in Figure 5. Generally, most DEGs were negatively correlated to the antibody responses. Only six (of 96) genes were highly (*R*^2^ ≥ 0.6) positively correlated across two or more antibodies tested including ENSGALG00000023172 (uncharacterized protein), ACTG2 (actin protein), SORBS2 (sorbin protein), CD180 (cell receptor involved in signaling), CXXC (transcription activator), and GNG10 (G protein).

### Commensal *E. coli* shedding in feces largely unaffected by vaccination

**Figure 6:**
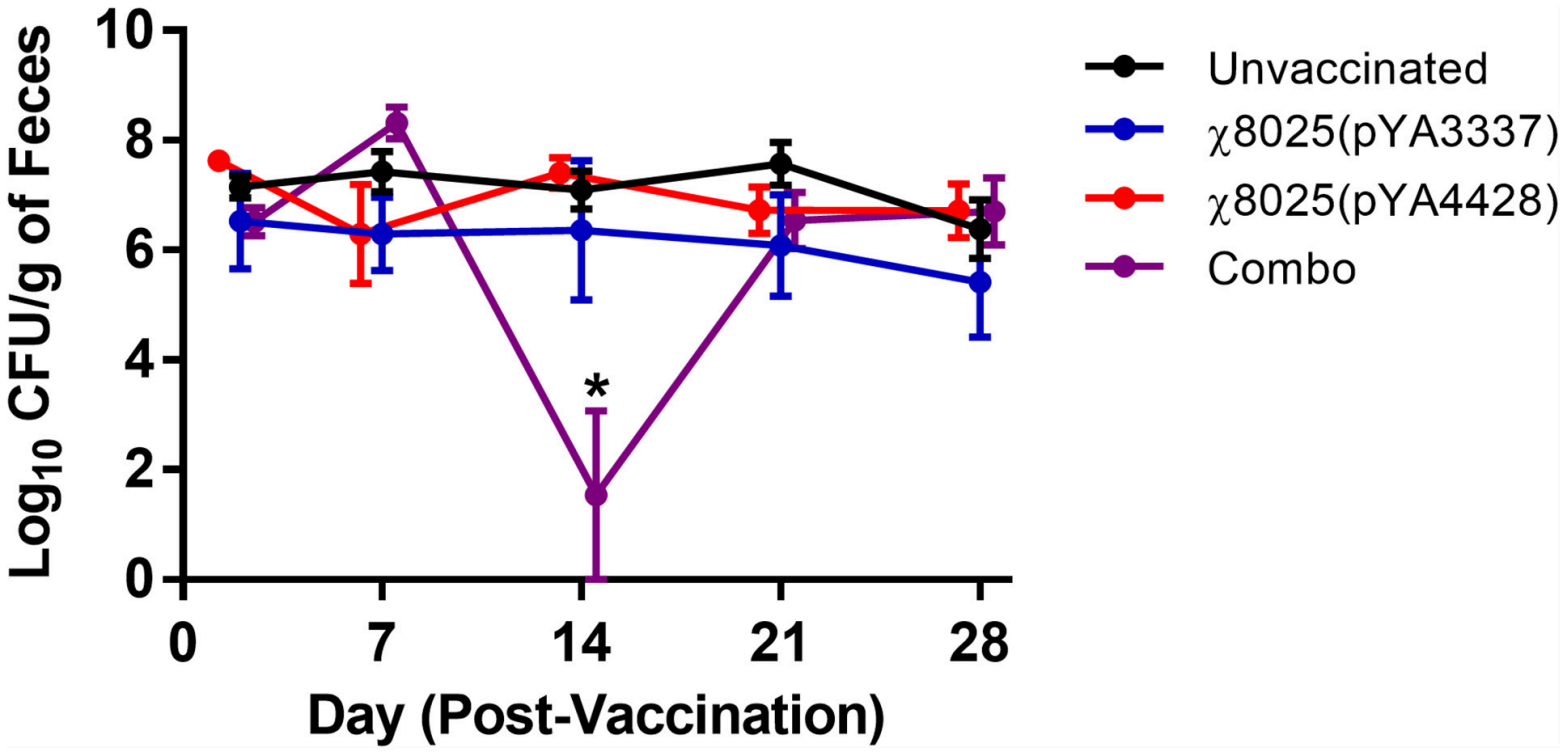
Fecal shedding of commensal *Escherichia coli* by chickens. Fecal samples (*n* = 3/group) were collected at approximately weekly intervals, weighed, homogenized in PBS, and plated on MacConkey agar for quantification of *E. coli*. Combo refers to the vaccine of χ8025(pYA3337) and χ8025(pYA4428). Statistically significant differences (*P* < 0.05) were represented by an asterisk (*) as determined by an ANOVA followed by Tukey's test for multiple means comparison. Points represent the mean and error bars represent the standard error of the mean.

To determine if vaccination had an impact on commensal *E. coli* levels, fecal samples were collected weekly and *E. coli* was quantified. On day 14, the Combo vaccine group shed significantly lower levels of *E. coli* in feces than other groups but recovered to a similar level as the other groups by day 21 (Figure 6). No other significant differences were detected between groups.

### Inhibitory effect of serum and intestinal wash samples

**Figure 7:**
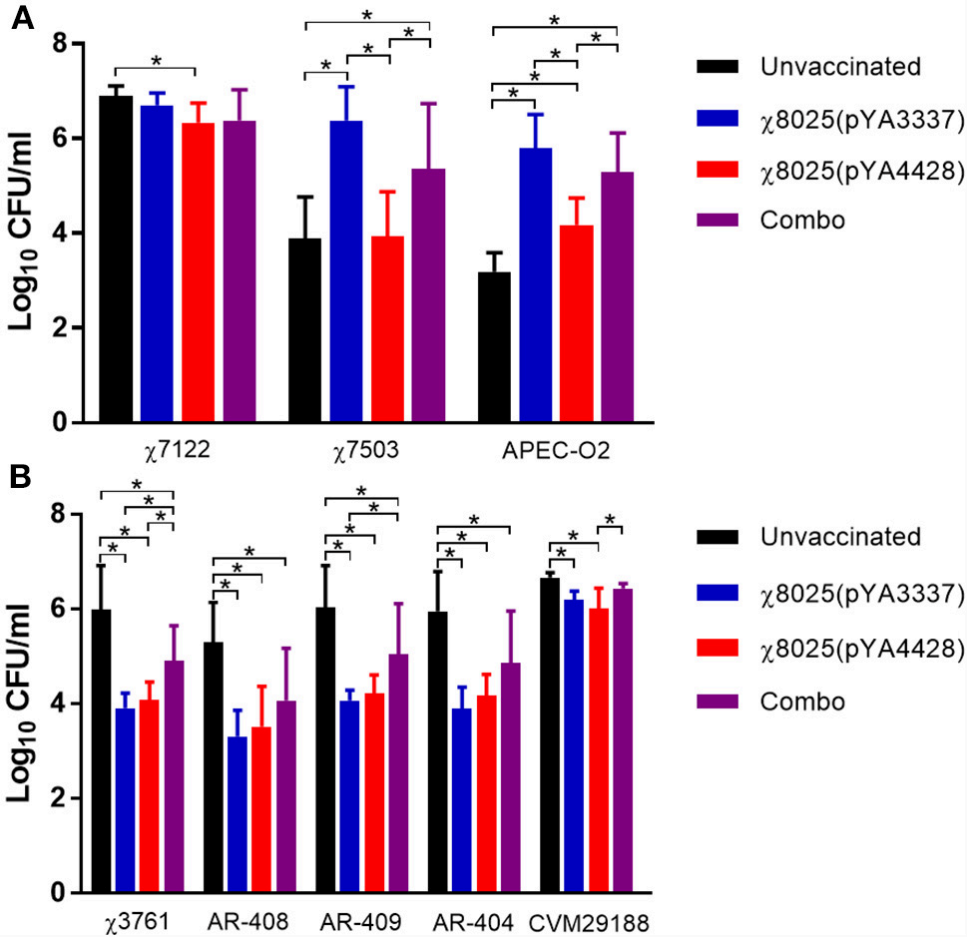
Bacterial inhibition of serum and intestinal wash samples from chickens. Bacterial strains were mixed 1:9 with pooled **(A)** serum or **(B)** intestinal wash samples and incubated at 40°C for 6 h. Combo refers to the vaccine of χ8025(pYA3337) and χ8025(pYA4428). An asterisk (*) represents a statistically significant difference (*P* < 0.05) as determined by an ANOVA followed by Tukey's test for multiple means comparison. Bars represent the mean and error bars represent standard deviations.

Bacterial strains were mixed with pooled serum or intestinal wash samples to determine if RASV immunization induced antibodies or other antimicrobial products that could influence bacterial levels. APEC strains cause systemic infections, and thus these organisms were mixed with serum samples. Overall, serum samples from vaccinated chickens had a variable response on bacterial inhibition of APEC strains (Figure 7). APEC strain χ7122 was significantly decreased (0.5 log CFU/ml) in serum samples of χ8025(pYA4428) vaccinated chickens compared with unvaccinated chickens (Figure 7A). χ7503 and APEC-O2 were significantly decreased in serum samples of unvaccinated and χ8025(pYA4428) compared with both χ8025(pYA3337) and Combo vaccinated chickens. In addition, APEC-O2 but not χ7503 was significantly reduced in serum samples from unvaccinated chickens compared with χ8025(pYA4428) vaccinated chickens.

Intestinal wash samples were tested with *S*. Heidelberg, *S*. Kentucky, and *S*. Typhimurium because they represent poultry-associated serotypes carried in the intestinal tract and are common causes of human infections. Generally, intestinal washes from vaccinated chickens inhibited (0.2–2.1 log CFU/ml) *Salmonella* strains (Figure 7B) compared to those from unvaccinated chickens. Four of five *Salmonella* strains (χ3761, AR-404, AR-408, and AR-409) were significantly (*P* < 0.05) reduced in intestinal washes of χ8025(pYA3337), χ8025(pYA4428), and Combo vaccinated chickens compared with unvaccinated chickens. In addition, χ3761 had significantly (*P* < 0.05) decreased levels in intestinal washes from χ8025(pYA3337) and χ8025(pYA4428) immunized chickens compared with Combo vaccinated chickens. AR-409 was significantly (*P* < 0.05) reduced in intestinal washes from χ8025(pYA3337) compared with Combo vaccinated chickens. CVM29188 was significantly (*P* < 0.05) reduced in intestinal washes from χ8025(pYA3337) and χ8025(pYA4428) compared with unvaccinated chickens, and in intestinal wash samples from χ8025(pYA4428) compared with Combo vaccinated chickens.

### Vaccine protection against APEC air sac challenge in chickens

In the air sac challenge, bacterial loads and gross lesion scores were evaluated to assess protection after RASV immunization. No birds died after challenge prior to planned euthanasia. There were no significant differences in bacterial loads or gross colibacillosis lesion scores between groups when challenged with APEC-O2 or χ7122 (Table 3). For the proportion of *E. coli* positive tissues, significant (*P* < 0.05) differences between groups were found only for APEC-O2 challenge. The liver and spleen of χ8025(pYA3337) immunized chickens and the lung of χ8025(pYA4428) immunized chickens had significantly (*P* < 0.05) fewer APEC-O2-positive samples than unvaccinated chickens.

**Table 3:** Mean lesion scores and level of avian pathogenic *Escherichia coli* in internal organs after immunization and challenge.

**Strain or immunization**	**Mean lesion score**		**Air sac**	**Blood**		**Heart**		**Liver**		**Lung**		**Spleen**	
	**Air sac**	**Heart and liver**	**Pro-portion positive**	**Pro-portion positive**	**Mean log_10_ CFU/ml**	**Pro-portion positive**	**Mean log_10_ CFU/g**	**Pro-portion positive**	**Mean log_10_ CFU/g**	**Pro-portion positive**	**Mean log_10_ CFU/g**	**Pro-portion positive**	**Mean log_10_ CFU/g**
**APEC-O2**													
Unvaccinated	0.8	0.9	5/9	3/9	1.3 ± 2.1	3/9	0.6 ± 1.0	4/9	1.5 ± 1.8	6/9	1.8 ± 1.4	4/9	1.6 ± 2.1
χ8025(pYA3337)	0.0	0.1	5/10	0/10	0.0 ± 0.0	1/10	0.3 ± 0.9	0/10^a^	0.0 ± 0.0	2/10	0.7 ± 1.5	0/10^a^	0.0 ± 0.0
χ8025(pYA4428)	0.0	0.2	6/10	1/10	0.3 ± 0.9	2/10	0.6 ± 1.3	2/10	0.7 ± 1.3	1/10^a^	0.3 ± 1.1	2/10	0.7 ± 1.5
Combo	0.3	0.4	4/9	3/9	1.0 ± 1.6	4/9	1.5 ± 1.9	5/9	2.0 ± 2.0	2/9	1.1 ± 2.2	4/9	1.7 ± 2.0
χ**7122**													
Unvaccinated	1.0	2.2	9/9	9/9	4.7 ± 0.7	8/9	3.2 ± 1.6	5/9	1.6 ± 1.6	8/9	2.4 ± 1.3	9/9	3.3 ± 1.3
χ8025(pYA3337)	0.9	2.4	12/12	12/12	4.9 ± 0.6	11/12	3.5 ± 1.7	10/12	2.4 ± 1.8	10/12	2.8 ± 1.7	12/12	3.5 ± 1.3
χ8025(pYA4428)	2.4	3.1	8/8	8/8	5.1 ± 0.7	8/8	4.7 ± 1.5	8/8	3.8 ± 2.0	8/8	3.8 ± 1.5	8/8	4.1 ± 1.9
Combo	1.7	2.5	10/10	9/10	3.7 ± 1.7	9/10	3.7 ± 1.7	7/10	1.9 ± 1.6	8/10	2.7 ± 1.6	10/10	3.5 ± 1.1

*Concentration data are represented by mean values ± standard deviation. Combo refers to the vaccine of χ8025(pYA3337) and χ8025(pYA4428)*.

a *Significant difference (P < 0.05) compared with unvaccinated chickens determined by a Fisher's exact test (two-tailed) for the proportion positive, or by an ANOVA followed by Tukey's multiple comparison test for mean bacterial loads*.

## Discussion

In this study, chicken responses to RASV strains were assessed phenotypically (antibody production) and genotypically (transcriptome); additionally, broad protection potential to *Salmonella* and APEC serotypes were tested *in vitro* and protection against APEC infection was tested *in vivo*. Multiple serotypes, virulence factors, and antibiotic resistance profiles present challenges to the prevention and treatment of APEC infections in chickens. Vaccines may represent a suitable option for the control of APEC infections. The RASV strain χ8025 was selected to deliver ECP because it has attenuating mutations (Δ*cya* Δ*crp*) of the avirulent Megan Vac 1 vaccine licensed in the United States for reduction of *Salmonella* in poultry and does not promote a *Salmonella* carrier state (37). ECP was selected as a candidate antigen because it is common among APEC, elicits an immune response in other ExPEC animal disease models, and plays a role in biofilm formation and systemic APEC infection in chickens (16, 31).

Previously, we found that ECP antigens were immunogenic and protective when subcutaneously injected in a mouse using a sepsis model (31). Similarly, high-levels of anti-*E. coli* IgY antibodies in chickens vaccinated with RASV strains were detected in the current study (Figure 1A). The ability of RASVs to elicit antibodies against IroN and IutA indicates cross-immunity between *Salmonella* and *E. coli* as demonstrated previously with RASV strain χ9558 (29). The elicitation of anti-IroN antibodies is expected as salmochelin *iroBCDEN* is found in *Salmonella* (38). In a previous study, colicin I receptor CirA and vitamin B12 transporter BtuB (both found in parental strain *S*. Typhimurium UK-1 χ3761) were found to share physical characteristics and amino acid sequence similarities to IutA (39). In addition, we have previously found that an RASV strain lacking ECP elicited antibodies against this antigen in mice as detected by ELISA but could not be confirmed by western blot (29). Thus, antibodies against ECP and IutA may have been detected in some chickens immunized without these factors due to common conformational epitopes shared between *Salmonella* and *E. coli* antigens.

The Combo group produced high-levels of anti-*E. coli* antibodies, one motivation for characterizing the spleen transcriptome of this group. We hypothesized that vaccination would result in changes in gene expression and that the spleen, a major immune organ for systemic host responses that previously responded to our recombinant antigen vaccine against APEC (36), would be an important site for modulating responses against APEC. Numerous studies have investigated changes to gene expression in the spleen of chickens challenged with *Salmonella* using a range of technologies from RT-qPCR to *high-throughput* sequencing (40–47). Collectively, these studies have established the importance of proinflammatory cytokines, cellular adhesions, and signaling in response to challenge with different serotypes of *Salmonella*. However, to our knowledge, only a single study exists on the spleen transcriptome investigating vaccination with a live *Salmonella* vaccine (45), indicating the novelty of the current study. In contrast to our study, Matulova et al. (45) used a *S*. Enteritidis ΔSPI1 mutant and identified 18 DEG comparing challenged unvaccinated to challenged vaccinated (45), whereas we identified 96 DEG in non-challenged samples. Because we focused on the comparison between vaccinated and unvaccinated samples that were not challenged, we may have identified more relevant DEG underlying mechanisms of protection. Previously, several immunoglobulin genes were upregulated (45), which is in agreement with the clustering results of immunoglobulin domain found in the current study (Table 2).

Overlap between our DEG by the Combo vaccination that includes an ECP producing strain and those found in APEC challenge may give insight into mechanisms of protection. Although some APEC strains have been investigated for ECP production under laboratory conditions (16), *in vivo* production patterns likely differ than those observed *in vitro* (48). Two studies used *high-throughput* sequencing to investigate the splenic response to APEC O1 challenge in chickens (49, 50). Overlapping pathways between these studies and the current study include T-cell receptor signaling and immunoglobulins, indicating the potential of our vaccine to prime the host for APEC challenge. In addition, the Combo vaccine upregulated 89 out of 96 DEG indicating a stimulatory mode of action of the vaccination. Two of the top 10 DEG were surfactant proteins A1 and A2 (Table S2). Although surfactant proteins are most often associated with pulmonary function, surfactant protein A stimulates macrophage chemotaxis and enhances binding of bacteria and viruses to alveolar macrophages in humans, with the equivalent being the network of phagocytes found in the avian lung (51). For the first time, we describe the enhanced expression of this protein in the spleen of vaccinated chickens, which may function as a biomarker for vaccine efficiency.

Two common themes amongst the immune-related DEG include pathogen recognition and host inflammation (*LOC419851, CLEC3B, TGFBR2*, and *MAPK11*) and adaptive immunity (*PIGR, MAL, THY1*, and *ISLR*). Regulation of gene expression at both the innate and adaptive immunity levels indicates immune system activation by our vaccine. Additionally, we identified *TGF-beta* receptor was upregulated in the Combo vaccine. TGF-beta has been implicated as an important cytokine in the spleen of chickens during infection with various pathogens (47, 52–54). By upregulating the receptor, the host may be more primed to quickly combat infections.

The functional annotation clustering revealed signaling as the most significantly impacted category (Table 2). Signaling is paramount to a successful vaccine as cells must communicate to initiate a robust response to infection. This category included *THY1* and *LOC419851*, immune-related genes, indicating that signaling may affect host immunity. The GO analysis revealed binding as a major category (Figure 3), affirming the importance of cellular communication during vaccination. Another enriched category was the immunoglobulin domain, involved in antibody-mediated adaptive immune response.

Protein-protein interactions are at the core of cellular response, including those involved in response to vaccination. In the current study, the strongest clustering (most connections) was between genes involved in cellular motility including various actin proteins (Figure 4). Actins are highly conserved proteins that are constitutively expressed in eukaryotic cells. Although mainly thought of in their motility function within muscle cells, actin polymerization in macrophages has been reported to be functionally linked to innate host immunity in defense against *Salmonella* infection (55). Intracellular pathogens, such as *Salmonella* rearrange host cytoskeleton during invasion. In another study, chickens infected with APEC showing mild pathology had an enriched actin regulation pathway compared to those with severe pathology scores, indicating that regulation of the actin cytoskeleton may be important for APEC resistance (56). Cytoskeleton rearrangements by the host may be a mechanism of protection by enabling intracellular killing of bacteria. Additionally, we identified numerous connections between these motility proteins with MAPK11, part of the well-studied signaling pathway. These MAP kinases are involved in all stages of immune responsiveness including innate and adaptive immunity (57). Here, we report increased expression of these motility-associated proteins (i.e., ACTA1, ACTA2, ACTG2, CNN3, MYH11, MYL9, and TAGLN) and MAPK11 in response to the Combo vaccination, which may be used as indicators of innate immune stimulation to provide better protection to the host. Future research should further characterize these protein-protein interactions.

A successful vaccine stimulates both innate and adaptive immunity (58) but how this occurs is not completely understood. Here we correlated antibody levels to gene expression in response to vaccination to give insight into the mechanism of protection. We found that in the Combo group the majority of DEG levels were negatively correlated with serum IgY anti-EcpA, -IroN, and -IutA antibody levels (Figure 5). Only six genes were highly positively correlated with 2 or more antibody responses. Interestingly, expression of *CD180* was very highly correlated (*R*^2^ = 0.91) with EcpA and IutA levels. The CD180 protein has been shown to activate B cells to quickly produce polyclonal Ig antibodies (59), which may contribute to the protection by our RASV. We hypothesize that the Combo vaccine largely stimulates innate immunity, at least at the timepoint tested here.

Most APEC vaccines in poultry have been designed using attenuated or inactivated *E. coli* strains (60–65) or with *E. coli* proteins (36, 66–69). Recently, RASVs have been used to deliver *E. coli* antigens including aerobactin receptor, CS31A surface antigen, *E. coli* O-antigens, P-fimbriae, and type 1 fimbriae to vaccinate against APEC in chickens (14, 15, 70–72). Collectively, these studies reported mixed results on protectiveness against APEC challenge. Vaccination with protein antigens alone presents problems with application in the hatchery and on farms such as possible local tissue damage, infection from contaminating organisms at the injection site, sanitation of injection equipment, and handling of animals that may result in stress (73). To overcome these problems, live vaccines can be delivered as a spray or in drinking water. Thus, this study used live RASV strains that were delivered orally. Because both pathogenic and non-pathogenic *E. coli* have *ecp*, the effect of vaccination on commensal *E. coli* levels in fecal samples was assessed. The only significant difference found was on day 14 for the Combo group but this finding could simply be an artifact of limited sampling. Overall, RASVs had a negligible effect on the concentration of commensal *E. coli* shed in the feces of chickens (Figure 6), which could be related to differences in ECP production between virulent and commensal *E. coli* as shown previously (16). In a recent study, the early microbiome of chickens <4-weeks-old was not impacted by oral inoculation with an attenuated *S*. Typhimurium strain (74). The current study confirmed an earlier finding that using a conserved *E. coli* antigen in a vaccine has no impact on commensal *E. coli* levels in the gastrointestinal tract (75).

*In vitro* assays were performed to assess the protective potential of RASV immunization using multiple APEC and *Salmonella* strains. Overall, a variable response was observed for APEC strains in serum samples from RASV immunized chickens, while intestinal wash samples from RASV immunized chickens strongly inhibited (0.2–2.1 log CFU/ml) *Salmonella* strains (Figure 7). The reason for the variability in serum sample results is unclear, but one possibility is that one or more chickens from the unvaccinated group had a transient increase in antimicrobial peptides (76) at time of collection that was effective in reducing χ7503 and APEC-O2 levels. Bacterial inhibition in intestinal wash samples was likely in part because of antibodies induced against conserved epitopes found across *S*. Heidelberg, *S*. Kentucky, and *S*. Typhimurium. In support of cross-protection, a study using a vaccine consisting of attenuated *S*. Enteritidis, *S*. Typhimurium, and *S*. Infantis demonstrated that heterologous protection can occur against *S*. Dublin and *S*. Hadar in chickens (77).

The protective ability of RASV strains was examined in an air sac challenge with APEC serogroups O2 and O78. APEC-O2 was found at lower concentrations in internal organs than χ7122, which is consistent with our earlier findings (2). For lesion scores and bacterial loads, no significant differences were detected between groups (Table 3). A few significant (*P* < 0.05) reductions in the number of APEC-O2-positive tissues were found for some RASV immunizations. These significant differences between RASV immunized and unvaccinated birds may be due to the strong IgY responses elicited after vaccination reported in this study. Although antibody responses are important, Sadeyen et al. (65) found that cell-mediated responses were also important in APEC vaccine-mediated protection in poultry. In a recent study, Powell et al. (78) found that RASVs induced distinct innate immune responses depending on the strain. Thus, χ8025(pYA3337) may have elicited protective cell-mediated responses against APEC that were not as strongly elicited in chickens immunized with χ8025(pYA4428) or Combo. In addition, whether the challenge strain produced ECP may have influenced the ability of the vaccine to protect against infection. Significant differences were found only with the ECP-positive strain APEC-O2; no significant differences were found with ECP-negative χ7122. Other studies reported failure of APEC vaccines to protect against homologous and/or heterologous challenge in poultry (60, 62, 68, 69, 79). In the current study, the lack of strong protection after vaccination may be due to the route of infection. Other studies assessing APEC infection used the intratracheal route of infection (13, 80, 81). In our study, using air sac inoculation that bypassed the trachea may have avoided important mucosal antibodies elicited by RASV immunization. Also, vaccine dose, antigen, length of challenge, and age at which vaccination and boost occur likely contribute to the amount of protection observed. Future studies should seek to optimize vaccine and challenge parameters.

In summary, RASV immunized chickens elicited anti-*E. coli* antibodies. This study was the first to assess the transcriptome of spleen tissue from RASV immunized chickens and revealed DEG between Combo vaccinated and unvaccinated chickens. By RNA-seq analysis, signaling was significantly impacted which included immune-related genes. Samples from RASV immunized chickens showed potential for *Salmonella* inhibition and some inhibition of APEC strain χ7122 *in vitro*. After APEC-O2 challenge, *E. coli* was detected in some vaccinated chickens significantly fewer number of times than unvaccinated chickens. However, bacterial loads and lesion scores were not significantly different. This study is an initial step toward developing improved therapeutics by using RASVs to stimulate the immune system to combat APEC infections in chickens. Future research is needed to optimize APEC antigens displayed by the RASV that could improve protective ability against APEC infection.

## Ethics statement

This study was carried out in accordance with the recommendations of the American Veterinary Medical Association Guidelines for Euthanasia, 2013 edition and Iowa State University Institutional Animal Care and Use Committee. The protocol (#1-16-8159-G) was approved by the Iowa State University Institutional Animal Care and Use Committee.

## Author contributions

MM conceived and designed the experiments. ZS, AV, GR, and MM performed the experiments. ZS, AV, and MM analyzed the data. MM contributed reagents, materials, analysis tools. ZS, AV, and MM wrote the paper. All authors read and approved the final manuscript.

### Conflict of interest statement

The authors declare that the research was conducted in the absence of any commercial or financial relationships that could be construed as a potential conflict of interest.

## Footnotes

^1^https://david.ncifcrf.gov

^2^http://pantherdb.org

^3^https://ncbi.nlm.nih.gov/geo/
