# Supplementary material for: Characterization of Spleen Transcriptome and Immunity Against Avian Colibacillosis After Immunization With Recombinant Attenuated Salmonella Vaccine Strains
Source: Front Vet Sci. 2018 Aug 21;5:198. doi: 10.3389/fvets.2018.00198 (PMC6113917; doi:10.3389/fvets.2018.00198)
Supplement: Supplementary file 1 [file Data_Sheet_1.PDF]

Supplementary Material

Characterization of Spleen Transcriptome and Immunity Against Avian Colibacillosis After Immunization with Recombinant Attenuated *Salmonella* Vaccine Strains

Zachary R. Stromberg, Angelica Van Goor, Graham A. J. Redweik, Melha Mellata*

***Correspondence:** Melha Mellata: mmellata@iastate.edu

1 Supplementary Tables

Table S1. RNA quality, sequences generated pre- and post-filtering, and percentage of genes expressed.

Sample[a]	RIN[b]	Total no. of sequences generated pre-filtering	Total no. of sequences post-filtering	Percentage sequences filtered	Percentage genes expressed[c]
PBS-1	10.0	47,410,288	40,886,627	16.0	89.5
PBS-2	10.0	42,208,335	32,681,152	29.2	88.5
PBS-3	9.9	50,921,120	44,697,842	13.9	89.2
PBS-4	9.8	40,280,157	33,449,298	20.4	88.9
Combo-1	9.6	51,793,489	44,184,612	17.2	89.7
Combo-2	9.6	48,987,712	38,210,822	28.2	90.9
Combo-3	9.8	50,579,636	44,197,841	14.4	89.8
Combo-4	9.1	56,414,858	46,448,593	21.5	90.0
Average	9.7	48,574,449	40,594,598	20.1	89.6

[a]Combo refers to the combination vaccine of χ8025(pYA3337) and χ8025(pYA4428).
[b]RIN, RNA integrity number.
[c]Calculated as the number of genes with counts $\geq$ 1 determined by HTSeq divided by the total number of annotated genes in the GalGal5 genome (14,468).

Table S2. Differentially expressed gene list.

Gene ID[a]	Name	Description	Fold change	Adj. *P* value
ENSGALG00000+A439 1:AA4401010825	Uncharacterized	N/A	426.3	0.03
ENSGALG00000021274	*LOC427778*	N/A	309.5	0.04
ENSGALG00000002503	*SFTPA2*	Surfactant protein A2	197.4	0.01
ENSGALG00000037632	*NKX2-1*	NK2 homeobox 1	158.0	0.04
ENSGALG00000014505	*FGFBP1*	Fibroblast growth factor binding protein 1	150.1	0.01
ENSGALG00000031496	*SPIK5*	Serine peptidase inhibitor, Kazal type 5	147.6	0.01
ENSGALG00000002496	*SFTPA1*	Surfactant protein A1	110.3	0.04
ENSGALG00000006469	*ATP12A*	ATPase H+/K+ transporting non-gastric alpha 2 subunit	110.0	0.01
ENSGALG00000015079	*UPK1B*	Uroplakin 1B	68.8	0.01
ENSGALG00000019719	*KRT19*	Keratin 19	55.7	0.02
ENSGALG00000011796	Uncharacterized	N/A	35.7	0.05
ENSGALG00000028560	*OC3*	Osteocalcin-like protein OC3	34.4	0.00
ENSGALG00000016473	*OSR1*	Protein odd-skipped-related 1	31.6	0.00
ENSGALG00000034868	*Otokeratin*	Keratin 7	26.9	0.01
ENSGALG00000000919	*PIGR*	Polymeric immunoglobulin receptor	25.6	0.01
ENSGALG00000003690	*KRT17*	Keratin, type I cytoskeletal 14	21.0	0.02
ENSGALG00000015147	*ALDH1A1*	Aldehyde dehydrogenase 1 family member A1	14.0	0.04
ENSGALG00000023953	*LOC419851*	Complement component 4 binding protein, alpha chain	13.0	0.05
ENSGALG00000005956	*ANXA8*	Annexin	9.7	0.04
ENSGALG00000026862	*CLDN1*	Claudin 1	9.5	0.01
ENSGALG00000040573	*FMO3*	Flavin containing monooxygenase 3	9.1	0.04
ENSGALG00000008552	*MAL*	Myelin and lymphocyte protein	7.3	0.03
ENSGALG00000003015	*SERPINF1*	Serpin family F member 1	4.3	0.03
ENSGALG00000011902	*TAGLN*	Transgelin	3.7	0.00
ENSGALG00000006751	*THY1*	T-helper 1 cell surface antigen	3.5	0.02
ENSGALG00000041634	*ACTG2*	Actin, gamma-enteric smooth muscle	3.0	0.00

ENSGALG00000011086	*ACTA1*	Actin, alpha skeletal muscle	2.6	0.00
ENSGALG00000011894	*CYP2D6*	Cytochrome P450 family 2 subfamily D member 6	2.4	0.00
ENSGALG00000001946	*RSPO1*	R-spondin 1	2.4	0.01
ENSGALG00000008085	*NEFH*	Neurofilament heavy	2.3	0.01
ENSGALG00000006190	*FHL1*	Four and a half LIM domains 1	2.2	0.00
ENSGALG00000000667	*EDN2*	Endothelin 2	2.1	0.04
ENSGALG00000039216	*COL6A2*	Collagen alpha-2(VI) chain	2.1	0.00
ENSGALG00000003261	*CAVIN1*	Caveolae associated protein 1	2.1	0.01
ENSGALG00000032220	Uncharacterized	N/A	2.1	0.00
ENSGALG00000029072	*NTN3*	Netrin 3	2.0	0.01
ENSGALG00000026055	*PALM*	Paralemmin	2.0	0.04
ENSGALG00000023472	*LOC415852*	Sulfotransferase	2.0	0.00
ENSGALG00000043829	*EXTL1*	Nxostosin like glycosyltransferase 1	2.0	0.01
ENSGALG00000006565	*GGT1*	Glutathione	2.0	0.00
ENSGALG00000030969	*CLEC3B*	C-type lectin domain family 3 member B	2.0	0.00
ENSGALG00000005974	*COL6A1*	Collagen alpha-1(VI) chain precursor	1.9	0.00
ENSGALG00000005422	*PDLIM1*	PDZ and LIM domain 1	1.9	0.01
ENSGALG00000013427	*SORBS2*	Sorbin And SH3 Domain Containing 2	1.9	0.04
ENSGALG00000035282	*IGF2*	Insulin-like growth factor II	1.8	0.00
ENSGALG00000029621	*SEMA3F*	Semaphorin-3F precursor	1.8	0.01
ENSGALG00000006471	*SLCO2A1*	Solute carrier organic anion transporter family member 2A1	1.8	0.00
ENSGALG00000034456	*PRELP*	Proline and arginine rich end leucine rich repeat protein	1.8	0.05
ENSGALG00000012055	*TGFBR2*	TGF-beta receptor type-2	1.8	0.02
ENSGALG00000002886	*NKX2-5*	NK2 homeobox 5	1.8	0.01
ENSGALG00000015193	*CCDC80*	Coiled-coil domain containing 80	1.8	0.05
ENSGALG00000042177	*GPR182*	G protein-coupled receptor 182	1.8	0.01
ENSGALG00000028567	*MYL9*	Myosin light chain 9	1.8	0.02
ENSGALG00000042628	Uncharacterized	N/A	1.8	0.04
ENSGALG00000000884	*CXXC5*	CXXC finger protein 5	1.7	0.04

ENSGALG00000021525	*ISLR*	Immunoglobulin Superfamily Containing Leucine Rich Repeat	1.7	0.04
ENSGALG00000003923	*COL6A3*	Collagen type VI alpha 3 chain	1.7	0.02
ENSGALG00000012023	*DACT1*	Dishevelled binding antagonist of beta catenin 1	1.7	0.00
ENSGALG00000002790	*ABLIM3*	Actin binding LIM protein family member 3	1.7	0.03
ENSGALG00000032499	Uncharacterized	N/A	1.7	0.04
ENSGALG00000033039	*ENG*	Endoglin	1.7	0.01
ENSGALG00000036125	*SHE*	Src homology 2 domain containing E	1.7	0.00
ENSGALG00000026983	*CHRD*	Chordin	1.7	0.05
ENSGALG00000025938	*KIAA0513*	KIAA0513	1.7	0.02
ENSGALG00000006343	*ACTA2*	Actin, alpha 2, smooth muscle, aorta	1.6	0.01
ENSGALG00000016215	*CXorf36*	Chromosome X open reading frame 36	1.6	0.03
ENSGALG00000002763	*FRZB*	Frizzled-related protein	1.6	0.01
ENSGALG00000024486	*C4orf48*	Chromosome 4 open reading frame 48	1.6	0.00
ENSGALG00000039628	*AFAP1L1*	Actin filament associated protein 1 like 1	1.6	0.02
ENSGALG00000037869	*NPR1*	Natriuretic peptide receptor 1	1.6	0.04
ENSGALG00000001561	*MXRA8*	Matrix-remodeling-associated protein 8	1.6	0.03
ENSGALG00000043499	*EGFL7*	EGF like domain multiple 7	1.6	0.03
ENSGALG00000037901	*EFEMP1*	EGF containing fibulin like extracellular matrix protein 1	1.6	0.04
ENSGALG00000006409	*PODXL*	Podocalyxin like	1.6	0.04
ENSGALG00000040993	*BAMBI*	BMP and activin membrane bound inhibitor	1.6	0.02
ENSGALG00000007000	*NR2F2*	Nuclear receptor subfamily 2 group F member 2	1.6	0.02
ENSGALG00000007582	*DPYSL3*	Dihydropyrimidinase like 3	1.6	0.04
ENSGALG00000040023	*MAPK11*	Mitogen-activated protein kinase 11	1.6	0.03

ENSGALG00000004184	*SPARC*	Secreted protein acidic and cysteine rich	1.5	0.03
ENSGALG00000028140	*CNTFR*	Ciliary neurotrophic factor receptor	1.5	0.05
ENSGALG00000014970	*FSTL1*	Follistatin like 1	1.5	0.01
ENSGALG00000014485	*LDB2*	LIM domain binding 2	1.5	0.04
ENSGALG00000010747	*FBLN5*	Fibulin 5	1.5	0.03
ENSGALG00000009957	*TIE1*	Tyrosine kinase with immunoglobulin like and EGF like domains 1	1.5	0.04
ENSGALG00000013605	*LRRC16A*	Leucine-rich repeat-containing protein 16A	1.5	0.05
ENSGALG00000005597	*CNN3*	Calponin 3	1.5	0.05
ENSGALG00000006520	*MYH11*	Myosin-11	1.5	0.03
ENSGALG00000012857	*DENND2A*	Rab guanyl-nucleotide exchange factor	1.4	0.04
ENSGALG00000028928	*LCAT*	Lecithin-cholesterol acyltransferase	1.4	0.03
ENSGALG00000006809	*POU2AF1*	POU class 2 associating factor 1	0.7	0.03
ENSGALG00000031117	*STK17A*	Serine/threonine kinase 17a	0.6	0.01
ENSGALG00000015683	*GNG10*	G protein subunit gamma 10	0.6	0.03
ENSGALG00000041344	*FABP5*	Fatty acid binding protein 5	0.6	0.05
ENSGALG00000023411	*CD180*	CD180 molecule	0.5	0.02
ENSGALG00000031794	*PDCD1LG2*	Programmed cell death 1 ligand 2	0.5	0.03
ENSGALG00000023172	Uncharacterized	N/A	0.5	0.00

[a]Ensembl gene ID based on genome build Galgal5.
