# Supplementary material for: Characterization of Spleen Transcriptome and Immunity Against Avian Colibacillosis After Immunization With Recombinant Attenuated Salmonella Vaccine Strains
Source: Front Vet Sci. 2018 Aug 21;5:198. doi: 10.3389/fvets.2018.00198 (PMC6113917; doi:10.3389/fvets.2018.00198)
Supplement: Supplementary file 2 [file Data_Sheet_2.PDF]

Supplementary Material

Characterization of Spleen Transcriptome and Immunity Against Avian Colibacillosis After Immunization with Recombinant Attenuated *Salmonella* Vaccine Strains

Zachary R. Stromberg, Angelica Van Goor, Graham A. J. Redweik, Melha Mellata*

***Correspondence:** Melha Mellata: mmellata@iastate.edu

1 Supplementary Figures

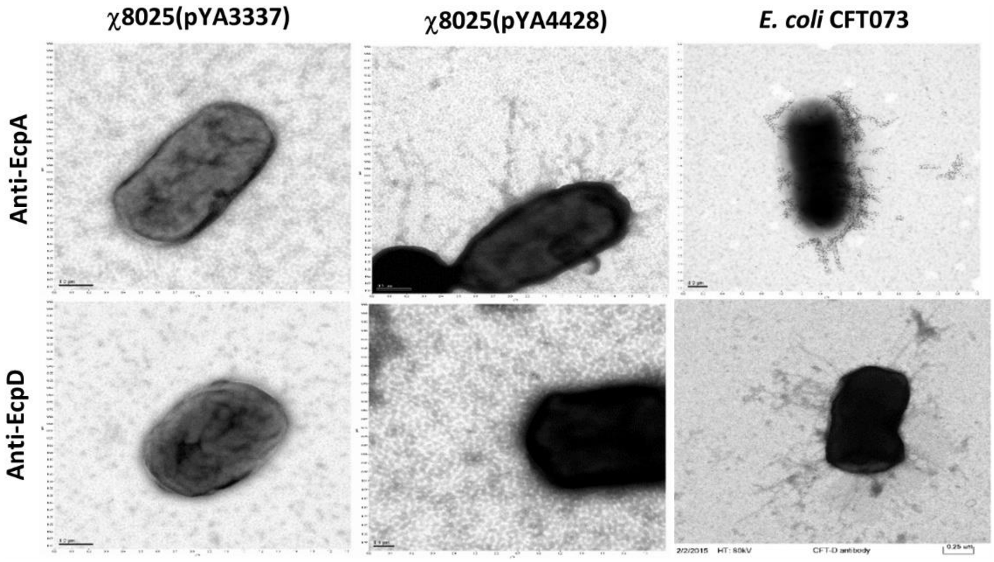

Figure S1. ***Escherichia coli*** **common pilus (ECP) synthesis on the surface of recombinant attenuated** ***Salmonella*** **vaccine (RASV) strain χ8025(pYA4428).** ECP synthesis is shown in RASV strain χ8025(pYA4428) carrying *ecp* genes and positive control *E. coli* CFT073. RASV strain χ8025(pYA337) lacking *ecp* genes was deficient in ECP synthesis.

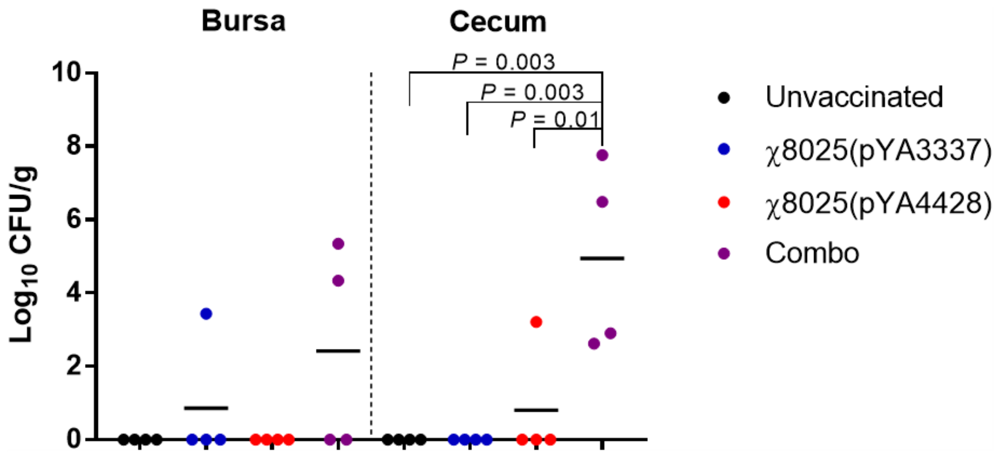

Figure S2. Recovery of recombinant attenuated *Salmonella* vaccine (RASV) strains from bursa tissue and cecal contents. Bursa tissue and cecal contents were aseptically collected, weighed, homogenized in PBS, and plated on MacConkey agar for quantification of RASV strains. Combo refers to the vaccine of χ8025(pYA3337) and χ8025(pYA4428). Statistically significant differences ($P < 0.05$) were determined by an ANOVA followed by Tukey's test for multiple means comparison. Each dot represents an individual animal.
